# Perinatal testosterone exposure and autistic-like traits in the general population: a longitudinal pregnancy-cohort study

**DOI:** 10.1186/1866-1955-4-25

**Published:** 2012-10-30

**Authors:** Andrew JO Whitehouse, Eugen Mattes, Murray T Maybery, Cheryl Dissanayake, Michael Sawyer, Rachel M Jones, Craig E Pennell, Jeffrey A Keelan, Martha Hickey

**Affiliations:** 1 Telethon Institute for Child Health Research, Centre for Child Health Research, University of Western Australia, 100 Roberts Road, Subiaco, Western Australia, 6008, Australia; 2 School of Psychology, University of Western Australia, 35 Stirling Hwy, Crawley, Western Australia, 6009, Australia; 3 School of Psychological Science, La Trobe University, Melbourne, Victoria, 3086, Australia; 4 Discipline of Paediatrics, University of Adelaide, Adelaide, South Australia, 5005, Australia; 5 School of Women’s and Infants’ Health, University of Western Australia, 35 Stirling Hwy, Crawley, Western Australia, 6009, Australia; 6 Department of Obstetrics and Gynaecology, University of Melbourne, Royal Women’s Hospital, Cnr of Flemington Road and Grattan Street, Parkville, Victoria, 3052, Australia

**Keywords:** Autism, Testosterone, Prenatal, Perinatal, Autistic-like traits

## Abstract

**Background:**

Increased prenatal testosterone exposure has been hypothesized as a mechanism underlying autism spectrum disorders (ASD). However, no studies have prospectively measured prenatal testosterone exposure and ASD. The current study sought to determine whether testosterone concentrations in umbilical cord blood are associated with a clinical diagnosis of ASD in a small number of children and with autistic-like traits in the general population.

**Methods:**

Umbilical cord blood was collected from 707 children. Samples were analyzed for total (TT) and bioavailable (BioT) testosterone concentrations. Parent report indicated that five individuals had a clinical diagnosis of ASD. Those participants without a diagnosis were approached in early adulthood to complete the Autism-Spectrum Quotient (AQ), a self-report measure of autistic-like traits, with 184 males (M = 20.10 years; SD= 0.65 years) and 190 females (M = 19.92 years; SD=0.68 years) providing data.

**Results:**

The BioT and TT concentrations of the five children diagnosed with ASD were within one standard-deviation of the sex-specific means. Spearman’s rank-order coefficients revealed no significant correlations between TT levels and scores on any AQ scale among males (rho range: -.01 to .06) or females (rho value range: -.07 to .01). There was also no significant association between BioT or TT concentrations and AQ scores among males (rho value range: -.07 to .08) or females (rho value range: -.06 to .12). Males were more likely than females to have ‘high’ scores (upper decile) on the AQ scale relating pattern and detail processing. However, the likelihood of a high score on this scale was unrelated to BioT and TT concentrations in both males and females.

**Conclusions:**

These findings indicate that testosterone concentrations from umbilical cord blood are unrelated to autistic-like traits in the general population. However, the findings do not exclude an association between testosterone exposure in early intrauterine life and ASD.

## Background

Autism Spectrum Disorder (ASD) is the collective term for neurodevelopmental conditions characterized by impairments in social interaction and communication, and a restricted range of activities and interests [1]. While the biological pathways contributing to ASD remain unclear, current consensus is for a multifactorial etiology, incorporating a constellation of genetic risk variants, perhaps interacting with environmental factors [2]. Males are between two and four times more likely to receive a diagnosis of ASD than females [3-5], and it has been suggested that this sex bias may provide clues as to the biological mechanisms subserving the condition [6,7]. Testosterone, the most biologically significant androgen, is a small lipophilic molecule that is able to pass the blood–brain barrier and cell membranes, and bind with androgen receptors found in the cytoplasm of cells. Once androgen receptor and testosterone (or its metabolite, dihydrotestosterone) molecules are bound, this structure is able to enter the nucleus, where it binds to DNA and affects transcription. The developing male fetus has considerably higher circulating testosterone levels compared to the female fetus [8], and animal studies have confirmed that prenatal testosterone exposure impacts upon fetal neurodevelopment both within and between sexes [9].

For many years, the primary way to investigate prenatal hormone exposure in humans was through an examination of ‘proxy measures’ of exposure, such as human finger-length patterns. Testosterone is thought to stimulate prenatal growth of the fourth finger while estrogen promotes growth of the second finger [10]. On average, males have a longer fourth digit relative to their second digit, while women have comparable second and fourth digit lengths [11]; a sex-difference that has been observed as early as the first trimester [12,13]. A lower 2D:4D ratio (indicating exposure to greater levels of testosterone) has been found among children with ASD and their relatives [14,15]. Further indirect evidence linking prenatal testosterone exposure and the ASD phenotype has emerged from studies of patient populations with excessive androgen exposure, such as females with Congenital Adrenal Hyperplasia (CAH). Most cases of CAH result from a genetic deficiency in the enzyme 21-hydroxylase, leading to an overproduction of adrenal androgens, such that female fetuses with CAH have prenatal androgen levels elevated to within or above the typical male range. Knickmeyer and colleagues [16] reported that females with CAH may have increased levels of autistic-like traits compared to non-affected siblings, particularly with regard to social difficulties and restricted interests, while another small study found an elevated rate of testosterone-related disorders among females with a clinical diagnosis of ASD, such as Polycystic Ovarian Syndrome and hirsutism [17]. Delayed menarche has also been linked with the ASD phenotype [18,19], although the association between prenatal testosterone exposure and age of menarche is inconsistent [20].

More direct evidence linking prenatal testosterone and the ASD phenotype has emerged from an ongoing longitudinal study of typically developing children for whom amniotic fluid was collected during the second prenatal trimester. Testosterone levels within the amniotic fluid samples were found to predict reduced eye contact at 12 months of age [21] and the quality of social relationships and level of restricted interests at age 4 and 8 years [22], as well as scores on a range of questionnaires measuring autistic-like behaviors, including the Quantitate CHecklist for Autism in Toddlers at age 18 to 24 months [23], and the Child Autism Spectrum Test and Autism-Spectrum Quotient- Child Version [24] between 6 and 10 years of age. Further analysis of the Autism-Spectrum Quotient-Child Version revealed amniotic fluid testosterone concentrations were positively associated with scores on all four subscales (Mind reading, Attention to detail, Social skills and Imagination). However, studies of this cohort have also limited their investigation to total testosterone levels only. Most circulating testosterone is bound to steroid hormone binding globulin (SHBG) and a smaller fraction to albumin. It is the 1% to 2% free fraction that is biologically active [25]. Measurements of total testosterone (TT), particularly during pregnancy when SHBG and albumin are increased, may not reflect free testosterone levels. Furthermore, studies measuring testosterone in amniotic fluid samples are limited by a certain sample demographic. Amniocentesis is performed only in high-risk pregnancies, most commonly for increased risk of fetal karyotypic abnormalities. Participant samples are not representative of the broader population of pregnant women, which may confound any study of prenatal testosterone exposure.

Umbilical cord blood presents another opportunity for estimating the prenatal hormone environment. Samples of cord blood can be obtained at delivery in both low and high risk pregnancies, and therefore randomly selected participant samples are more likely to be representative of the general population. A number of studies have reported higher testosterone levels in cord blood samples from male versus female fetuses [26,27], and thus these samples are thought to reflect fetal circulation during late gestation. A limitation of this approach is that testosterone levels in cord blood may not reflect concentrations during the earlier stages of pregnancy. Gestational weeks 8 to 24 have traditionally been regarded as a ‘sensitive period’ for the maximal effects of sex steroids on neurodevelopment [9]. While second trimester amniotic fluid samples are able to measure this critical window, umbilical cord samples are not. However, there is increasing recognition that there may be multiple sensitive periods, and animal studies have found that different neural regions may be affected by hormones at different times throughout prenatal development [28]. Investigations of the Western Australian Pregnancy Cohort (Raine) Study have provided evidence that umbilical cord blood can provide important data on testosterone and its effect on postnatal development, finding a 2.5-fold increase in the rate of language delay among males with high levels of cord-blood testosterone [29].

Here, we report a follow-up of the Raine cohort, representing the first investigation of the prospective relationship between umbilical cord testosterone concentrations and autistic characteristics. Cord blood was available for a small number of individuals with a clinical diagnosis of ASD, and a measure of autistic-like traits was obtained in early adulthood on the remainder of the available cohort. The measure of autistic-like traits was treated as a continuous variable, but was also dichotomized at the threshold of the upper decile, in order to determine whether perinatal testosterone levels were associated with high levels of autistic-like traits in particular. We hypothesized that increased concentrations of testosterone from cord blood would be associated with increased ASD-like traits in the whole sample, and that those individuals with a clinical diagnosis of ASD would have particularly high concentrations of umbilical cord testosterone.

## Methods

### Participants

The Raine Study is a longitudinal investigation of women between 16 and 20 weeks pregnancy from the public antenatal clinic at King Edward Memorial Hospital or surrounding private clinics, between May 1989 and November 1991. Approximately 100 unselected antenatal patients per month were enrolled during this period, with a final sample of 2,900 women. The inclusion criteria were a gestational age between 16 and 20 weeks, English language skills sufficient to understand the study demands, an expectation to deliver at King Edward Memorial Hospital, and an intention to remain in Western Australia to enable future follow-up of their child [30]. By the end of the recruitment period, 2,868 live births (96%) were available for follow-up. Participant recruitment and all follow-ups of the study families were approved by the Human Ethics Committee at Kind Edward Memorial Hospital and/or Princess Margaret Hospital for Children in Perth. Informed consent was obtained from all mothers and offspring who participated in this study.

### Testosterone analysis

Mixed arterial and venous umbilical cord blood was obtained at the birth of 861 singleton deliveries (selected randomly). Blood samples were immediately centrifuged, plasma isolated and then stored at −80°C without thawing. Detailed sequence analysis of DNA obtained from ten maternal-child pairs confirmed that the cord blood samples were not contaminated by maternal blood. In January 2010, these serum samples were thawed and analyzed for androgen content. TT was measured by liquid chromatography-tandem mass spectrometry (LC-MS/MS) after solvent extraction as described in detail by Keelan *et al*. [27]. The limit of quantitation was 0.025 ng/ml (0.08 nmol/L). Inter-batch variation was low at 6% to 11% (n = 24); recovery from cord serum was 93% to 98%. SHBG was measured by ELISA using a commercial kit (IBL International, Hamburg, Germany) according to the manufacturer’s instructions. All samples were measured in duplicate by a single operator using assay kits from the same batch. The inter-assay imprecision was <4.5% (n = 25). Intra-assay variation was 5.2% (n = 861). Samples with an initial replicate coefficient of variation (CV) of >10% were reanalyzed. BioT (nmol/L), representing the fraction of total testosterone either free (unsequestered by SHBG) or bound to serum albumin, was calculated using the formula: BioT = [free testosterone] + [albumin-bound testosterone] (19). Free testosterone was calculated using the empirical method and formula described by Sartorius *et al*. [31]. Albumin levels were adjusted using published reference values to take into account the decrease in serum albumin concentrations with gestational age [32]. For the current study, TT and BioT were the predictor variables of interest.

### ASD diagnosis

At the 5-, 8-, 10-, 14- and 17-year follow-ups of the Raine cohort, parents were asked whether their child had ever received a diagnosis of ASD by a health professional. Diagnosis of these conditions in Western Australia mandates consensus by a team comprising a pediatrician, psychologist and speech-language pathologist under Diagnostic and Statistical Manual of Mental Disorders, 4th Edition (DSM-IV), guidelines [1].

### Autism-spectrum quotient (AQ)

At age 19 to 20 years, the cohort was invited to complete the Autism Spectrum Quotient (AQ). Adults with a known diagnosis of any intellectual disability or ASD were not asked to complete the AQ due to ethical concerns. The AQ is a self-report questionnaire that provides a quantitative measure of autistic-like traits in the general population [33]. Individuals are provided with 50 statements and asked to indicate on a four-point scale how well that statement applies to them (strongly agree, agree, disagree, strongly disagree). Items were scored according to the original procedure as well as an alternative method. The original procedure outlined in Baron-Cohen *et al*., allocates a score of 1 to a response indicating an autistic-like trait (strongly agree/agree or strongly disagree/disagree) and 0 to a response non indicative of an autistic-like trait. The statements are divided into five subscales - Social Skills, Communication, Attention Switching, Attention to Detail, Imagination – of 10 items each. Items within each subscale are then summed to provide a quantitative measure of that particular autistic-like trait, with higher scores denoting increased symptomatology. A Total AQ is calculated by tallying the scores from the five subscales. The Total AQ is known to have good test-retest reliability (r = 0.7), and validation studies have found that scores in the general population follow a normal quantitative distribution [33,34].

The AQ was also scored according to an alternative procedure. Factor analyses of the AQ in several countries have consistently identified three clear factors, relating to Social Ability, Attention to Detail/Patterns, and Communication/Mind Reading. There is minimal difference between the items pertaining to these subscales and those reported in other factor analyses. In the current study, we divided items into the subscales identified in a study of Western Australian adults [35], who were highly similar to the sample under investigation here: Social Skills, Details/Patterns and Communication/Mindreading. The items were scored on a scale ranging from 1 to 4, based on previous research that this scoring method retains more information about responses than the dichotomous scoring first proposed for this instrument [35-37]. For the current data set, internal reliability for the scales ranged from moderate (Communication/Mind-reading: α = .63) to good (Details/Patterns: α = .78) and excellent (Social Skills: α = .85).

### Sample characteristics

A range of variables was investigated to determine whether the individuals with both cord blood testosterone and AQ data were representative of the broader cohort with BioT data. These included sociodemographic factors recorded at 18 weeks’ pregnancy (maternal age at conception, maternal education, family income); antenatal variables recorded at 34 weeks’ pregnancy (maternal smoking and alcohol consumption during pregnancy); and obstetric variables recorded at birth (gestational age, offspring gender, parity, Apgar scores five minutes after birth). A further variable, proportion of optimal birthweight, provided a measure of appropriateness of fetal growth. This variable is calculated as a ratio of the observed birth dimension to the optimal birth dimension for that individual neonate [38].

### Statistical analysis

Previous studies have identified sex-specific effects of perinatal testosterone exposure [29], and therefore all analyses were conducted separately for males and females. The first analysis examined concentrations of umbilical cord TT and BioT levels among the children with a parent-reported diagnosis of ASD. Raw scores were also converted to sex-specific Z-scores to enable a comparison of individual data points with the distribution of the broader sample.

The analyses then turned to the analysis of individuals with both testosterone and AQ data. For these data, we sought first to determine whether these participants were representative of the broader Raine Study cohort. TT/BioT concentrations and AQ scores were then investigated as both continuous and categorical data. AQ data were analyzed in accordance with the original scoring procedure [33], as well as the alternative scoring procedure outlined in the Methods [35]. With regards to the continuous variables, independent-samples t-tests examined whether there were sex differences in AQ scores. Given evidence that males score higher than females on the AQ and its subscales, one-tailed tests were conducted [33,37]. Our hypothesis is that early testosterone exposure is one potential reason for between sex-differences in autistic-like characteristics. As such, only those AQ scales in which there was a significant sex-difference in scores were examined further. For these scales, we conducted correlations between BioT and TT concentrations from umbilical cord blood and AQ scores. Any significant correlation was then followed up using hierarchical multivariate linear regression. Covariates that showed a correlation with the outcome variable (score on relevant AQ scale) at the level of *P* <.2 were included in the first block using the enter method, while concentrations of the predictor variable (TT or BioT) were added in the second block. For all analyses, an alpha level of 0.05 was adopted to indicate statistical significance.

We then investigated the data when expressed categorically to determine whether early testosterone exposure was associated with high scores on the AQ and its subscales. ‘High’ scores were defined as scores in the upper decile of a particular scale (to the nearest whole number). Chi-square analyses examined whether there were sex differences in the proportion of males and females with high scores on AQ each scale (both original and alternative scoring). Again, only those scales in which there was a significant sex-difference were examined further. For these scales, chi-square analyses were conducted separately for each sex between sex-specific quartiles of testosterone concentrations and high scores on the particular AQ scale. Any significant difference on any scale was followed up with multivariate logistic regression, following the same three-step procedure outlined for the multivariate linear regression. The alpha level for all analyses was *P* <.05.

## Results

### Clinical ASD

Among the 861 children with available BioT data, 707 (82.1%) provided diagnostic data at any of the 5-, 8-, 10-, 14- or 17-year follow-ups. Parent report indicated that 5 of the 707 (0.71%) children had received a clinical diagnosis of ASD. One male and one female had been diagnosed with Autistic Disorder, and three males had been diagnosed with Asperger’s Disorder. TT (BioT) concentrations for males with ASD ranged from 0.25 to 0.70 nM/L (0.079 to 0.22 nM/L), which compared to a mean in the broader male sample of 0.53 nM/L (0.14 nM/L) and a standard deviation of 0.36 nM/L (0.11 nM/L). The cord blood TT (BioT) concentration for the female with Autistic Disorder was 0.17 nM/L (0.05 nM/L), which was less than the mean of the broader female sample of 0.30 nM/L (0.09 nM/L) and standard deviation of 0.19 nM/L (0.07 nM/L). Table 1 presents sex-specific Z-scores for the TT and BioT concentrations of the individuals with ASD. All Z-scores were between −1 and 1, indicating that BioT concentrations were within one standard deviation of the sex-specific means.

**Table 1:** Testosterone concentrations from umbilical cord blood of the individuals with ASD

		**Bioavailable testosterone**		**Total testosterone**	
	**Sex**	**Raw value (nM/L)**	**Sex-specific Z score**	**Raw value (nM/L)**	**Sex-specific Z score**
Autism 1	Male	.35	−0.50	.10	−0.41
Autism 2	Female	.17	−0.68	.05	−0.50
Asperger 1	Male	.70	0.47	.22	0.70
Asperger 2	Male	.25	−0.78	.08	−0.62
Asperger 3	Male	.35	−0.50	.10	−0.44

ASD, autism spectrum disorder.

### Autistic-like traits

Among the 707 individuals who had provided some postnatal diagnostic data, 184 of 365 (50.4%) males and 190 of 342 (55.6%) females also completed the AQ. Although there was a statistically significant sex-difference in age at the time of AQ completion, t(372) = 2.73, *P* = .01, the absolute difference between males (M = 20.10 years; SD = 0.65 years) and females (M = 19.92 years; SD = 0.68 years) was small (Cohen’s *d* = 0.27). Table 2 presents the sociodemographic characteristics of these participants in comparison to the remainder of individuals with BioT data available. Chi-square analyses revealed that participants who did not complete the AQ had mothers who were younger, less educated, more likely to smoke cigarettes and have a family income below the poverty line ($AUD24,000) at the time of pregnancy. However, there was no difference between individuals who did and did not complete the AQ in gestational age at birth, proportion of optimal birthweight, and parity or Apgar scores five minutes after birth. Furthermore, there was no significant difference in BioT levels between those who did and did not contribute AQ data for both males, t(340) = 0.67, *P* = .51, and females, t(362) = 1.40, P = .16. Therefore, while the current sample was representative of the broader Raine cohort in terms of obstetric factors, they were exposed to a more socially advantageous environment.

**Table 2:** Characteristics of participants with testosterone data, who did or did not complete the Autism-Spectrum Quotient (AQ)

	**Completed AQ (n = 374)**		**Did not complete AQ (n = 332)**		
**Categorical variables**	**N**	**n (%)**	**N**	**n (%)**	***P*value**
Maternal age at birth	366		320		<.01
<20		19 (5.2)		32 (10.0)	
20-24		65 (17.8)		73 (22.8)	
25-29		91 (24.9)		107 (33.4)	
30-34		120 (32.8)		76 (23.8)	
35+		71 (19.4)		32 (10.0)	
Maternal education at pregnancy	366		320		.01
Completed secondary school		156 (42.6)		104 (32.5)	
Did not complete secondary school		210 (57.4)		216 (67.5)	
Family income below poverty line	364		318		<.01
Yes		115 (31.6)		149 (46.9)	
No		249 (68.4)		169 (53.1)	
Maternal smoking in pregnancy	366		320		.01
None		293 (80.1)		223 (69.7)	
1 to 10 cigarettes daily		42 (11.5)		56 (17.5)	
11+ cigarettes daily		31 (8.5)		41 (12.8)	
Maternal alcohol intake during pregnancy	366		320		.01
None		199 (54.4)		212 (66.3)	
Once a week or less		143 (39.1)		88 (27.5)	
Several times a week or more		24 (6.6)		20 (6.3)	
Gestational age	366		320		.36
< 32 weeks		3 (0.8)		7 (2.2)	
32 to 37 weeks		64 (17.5)		48 (15.0)	
38 to 40 weeks		231 (63.1)		210 (65.6)	
> 40 weeks		68 (18.6)		55 (17.2)	
Proportion of optimal birthweight	372		332		.41
<90		117 (31.5)		96 (28.9)	
90 to 110		208 (55.9)		183 (55.1)	
>110		47 (12.6)		53 (16.0)	
Sex	374		332		.14
Male		190 (50.8)		181 (54.5)	
Female		184 (49.2)		151 (45.5)	
Apgar scores	366		319		.94
Generally normal		353 (96.4)		308 (96.6)	
Fairly low		13 (3.6)		11 (3.4)	
Critically Low		0 (0)		0 (0)	
Parity	374		332		.51
1		167 (44.7)		159 (47.9)	
2		121 (32.4)		95 (28.6)	
>2		86 (23.0)		78 (23.5)	

### Continuous data

Table 3 presents descriptive statistics for predictor (TT and BioT) and outcome (AQ scores) variables for males and females. Independent-samples t-tests confirmed that males had significantly greater levels of TT and BioT compared to females (*P* <.01). Analyses of the original AQ scales revealed only one significant sex difference, which was for the Imagination subscale, where males had significantly higher scores than females (*P* = .01). Analyses of the alternative AQ scales found that males scored higher than females on the Total AQ (*P* = .02), as well as the Social Skills (*P* = .04) and the Details/Patterns subscales (*P* <.01). There were no statistically significant differences between the scores of males and females on the Total, Social Skills, Communication, Attention Switching and Attention to Detail scales scored according to the original procedure, and no difference on the Communication/Mindreading scale scored according to the alternative procedure.

**Table 3:** Mean (SD) of testosterone and Autism-Spectrum Quotient (AQ) scores

	**Males (N = 184) M (SD)**	**Females (N = 190) M(SD)**	***P*value**	**Cohen’s*d***
Total testosterone (nM/L)	0.49 (0.26)	0.27 (0.15)	<.01	1.03
Bioavailable testosterone	0.13 (0.06)	0.08 (0.04)	<.01	0.98
AQ original scoring				
Total	15.10 (4.78)	14.70 (5.31)	.23	0.08
Social Skills	1.51 (1.67)	1.68 (1.76)	.18	0.09
Communication	2.03 (1.57)	1.98 (1.68)	.39	0.03
Attention Switching	3.83 (1.90)	3.78 (1.98)	.32	0.03
Attention to Detail	5.34 (2.10)	5.24 (1.99)	.37	0.05
Imagination	2.39 (1.57)	2.03 (1.48)	.01	0.24
AQ alternative scoring				
Total	104.46 (12.23)	102.11 (10.53)	.02	0.19
Social Skills	24.66 (5.58)	23.64 (5.85)	.04	0.18
Details/Patterns	20.16 (4.63)	18.74 (4.35)	<.01	0.32
Communication/Mindreading	15.01 (2.88)	15.42 (3.50)	.22	0.12

**Figure 1:**
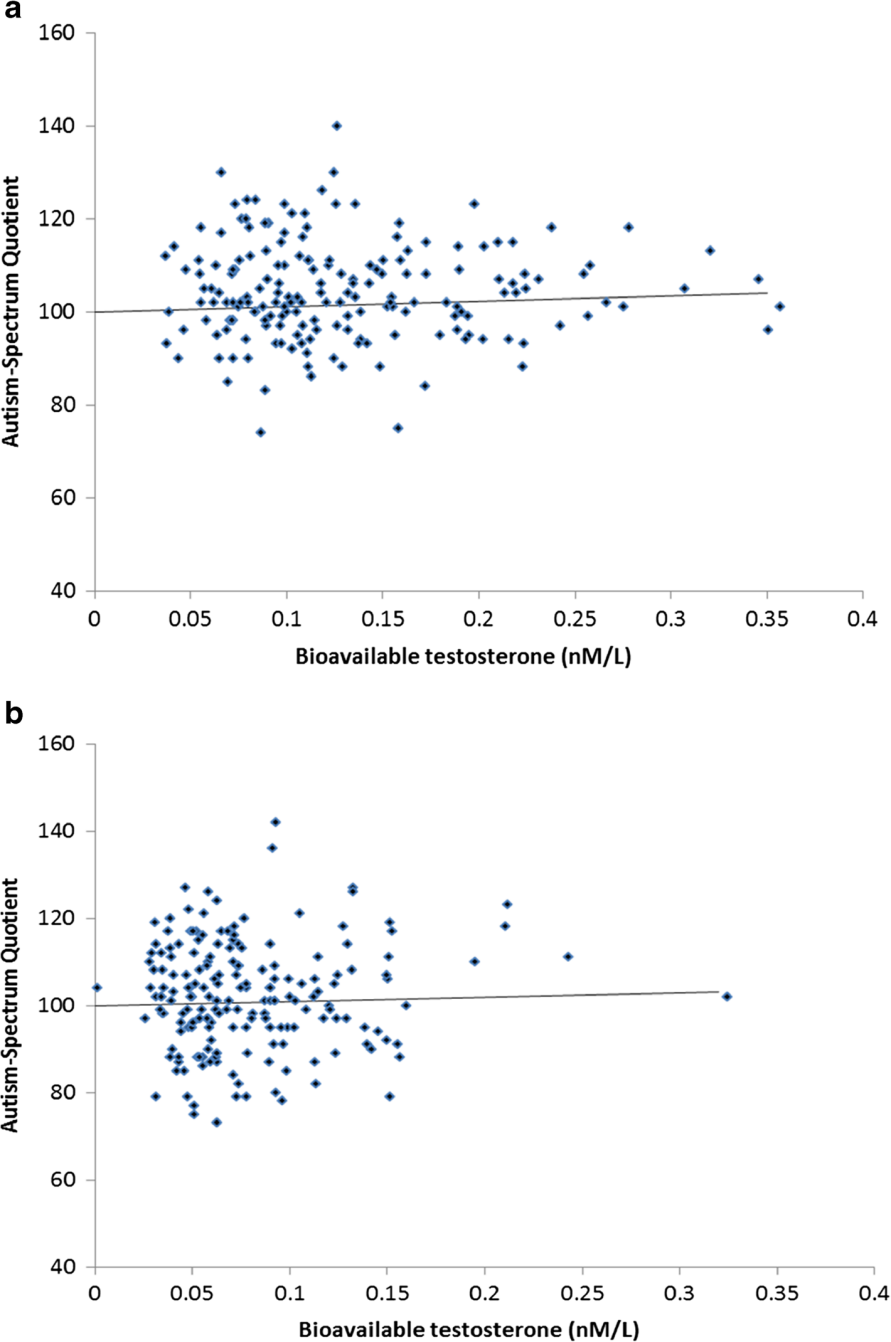
Scatterplots (and fit lines) showing the associations between bioavailable testosterone from umbilical cord blood (BioT) and total AQ scores in early adulthood in males (Figure 1a) and females (Figure 1b).

Testosterone concentrations were positively skewed (skewness statistic greater than 1 for BioT and TT for both males and females), and, therefore, Spearman’s rank-order correlations were conducted (Table 4). There were no significant correlations between predictor and outcome variables at the level of *P* <.05 for either sex. Spearman’s rho ranged from -.07 to .08 for males, and -.06 to .12 for females. Figures 1a and 1b present the raw data between BioT concentrations and the Total AQ score (alternative scoring) for males and females, respectively. Given the small effects observed and the lack of statistical significance, multivariate linear regression analyses were not conducted.

**Table 4:** **Spearman’s correlations (*P*value) between testosterone concentrations and scores on the Autism-Spectrum Quotient (AQ)**^**a**^

	**1**	**2**	**3**	**4**	**5**	**6**
1. Total testosterone	1	.94 (<.01)	-.04 (.60)	.01 (.89)	.05 (.47)	.06 (.40)
2. Bioavailable testosterone	.91 (<.01)	1	-.07 (.35)	.02 (.80)	.07 (.36)	.06 (.46)
3. Original: Imagination	.09 (.23)	.12 (.11)	1	.42 (< .01)	.14 (.06)	.02 (.82)
4. Alternative: Total AQ	-.03 (.68)	.01 (.85)	.40 (<.01)	1	.67 (<.01)	.37 (<.01)
5. Alternative: Social Skills	-.06 (.45)	.01 (.95)	.19 (.01)	.70 (<.01)	1	.05 (.47)
6. Alternative: Details/Patterns	-.05 (.49)	-.01 (.87)	.03 (.68)	.48 (<.01)	.20 (.01)	1

^a^Male correlations (n = 184) are above the diagonal, and female correlations (n = 190) are below the diagonal.

### Categorical data

High score thresholds (upper decile) for the various AQ scales are shown in Additional file 1. Chi-square analyses revealed a sex difference in the proportion of individuals with high scores on one AQ scale only (Table 5). Males (13%) were significantly more likely than females (5.3%) to have a high score on the Details and Patterns subscale (alternative scoring), *Χ*^2^ = 6.85, df = 1, *P* <.01. No other sex differences were observed.

**Table 5:** Number (%) of participants with ‘high’ scores on Autism-Spectrum Quotient (AQ) scores

	**Males (N = 184) n (%)**	**Females (N = 190) n (%)**	***P*value**	***Cramér*’*s Phi***
AQ original scoring				
Total	20 (10.9)	22 (11.6)	.83	0.01
Social Skills	8 (4.3)	15 (7.9)	.15	0.07
Communication	17 (9.2)	16 (8.4)	.78	0.08
Attention Switching	13 (3.1)	14 (7.4)	.91	0.01
Attention to Detail	34 (18.5)	27 (14.2)	.26	0.06
Imagination	16 (8.7)	10 (5.3)	.19	0.07
AQ alternative scoring				
Total	24 (13.0)	18 (9.5)	.27	0.06
Social Skills	18 (9.8)	17 (8.9)	.78	0.01
Details/Patterns	24 (13.0)	10 (5.3)	<.01	0.14
Communication/Mindreading	17 (9.2)	16 (8.4)	.78	0.08

Quartile markers for BioT concentrations among males were <0.083 nM/L (n = 46), 0.084 to 0.112 nM/L (n = 46), 0.113 to 0.162 nM/L (n = 46), and >0.162 nM/L (n = 46) for Quartiles 1 to 4, respectively. For females, markers were <0.049 nM/L (n = 47), 0.050 to 0.068 nM/L (n = 48), 0.069 to 0.098 nM/L (n = 48) and >0.99 nM/L (n = 47) for Quartiles 1 to 4, respectively. Given the very high correlations between TT and BioT levels, quartiles for TT were not analyzed. Chi-square analyses (Table 6) found that the proportion of high scores on the Details and Patterns subscale of the AQ (alternative scoring) did not significantly vary as a function of BioT quartiles in males, *Χ*^2^ = 1.15, df = 3, *P* <.77, and females, *Χ*^2^ = 6.07, df = 3, *P* = .11. The null results meant that no logistical regression analyses were conducted.

**Table 6:** The number (%) of participants with high scores on the Details and Patterns scale according to bioavailable testosterone concentrations

	**Bioavailable testosterone concentrations**					
	**Quartile 1 (lowest)**	**Quartile 2**	**Quartile 3**	**Quartile 4 (highest)**	***P*value**	***Cramér***’***s Phi***
Males	6 (13.0)	5 (10.9)	5 (10.9)	8 (17.4)	.77	.08
Females	2 (4.3)	0 (4.2)	2 (4.2)	5 (10.6)	.11	.15

### Sample attrition

Sample attrition was biased towards variables indexing lower socioeconomic strata. As a final set of *post*-*hoc* analyses, we examined whether Total AQ scores (original scoring procedure) in the current sample varied according to these factors. Independent-samples t-tests found that Total AQ scores were significantly higher for those adults whose mother was living below the poverty line during pregnancy (below poverty line: M = 15.82, SD = 5.09; above poverty line: M = 14.45, SD = 5.08; *P* = .02), and whose mother had not completed secondary school at the time of pregnancy (not completed secondary school: M = 15.53, SD = 4.73; completed secondary school: M = 14.25 , SD = 5.35; *P* = .01). However, analysis of variance (ANOVA) found that AQ scores in the current sample did not differ as a function of maternal age at conception, F(4, 361) = 0.50, *P* = .76, and maternal smoking during pregnancy, F(2, 363) = 1.11, *P* = .33.

## Discussion

Exposure to increased levels of prenatal testosterone has received increasing attention as a possible biological mechanism contributing to ASD. However, ASD is first recognized through behavioral symptoms during early childhood, and, therefore, data on the prenatal hormone environment are rare. This is the first report of the relationship between testosterone concentrations in umbilical cord blood and the ASD phenotype. Five children in the current sample had received a clinician-based diagnosis of ASD. Four of the five cases had TT and BioT levels lower than the sex-specific BioT means of the broader cohort, and all cases were within one standard deviation of these means. A measure of autistic-like traits, the AQ, was collected from 374 members of the cohort without ASD. Males scored higher than females on the three scales, but none of these were significantly correlated with early testosterone measurements (in males or females). Males were more likely than females to have ‘high’ scores on the Details and Patterns subscale of the AQ (but no other subscale). However, again, this scale was found to be unrelated to early testosterone concentrations. While the small number of individuals with a clinical diagnosis of ASD evokes caution in the interpretation of these data, we suggest that the current findings indicate that testosterone concentrations measured at the time of birth are not associated with autistic-like traits in the general population.

The Cambridge Fetal Testosterone Project has provided the most direct evidence linking prenatal testosterone exposure and ASD, reporting associations between testosterone levels in amniotic fluid and a range of autistic-like traits during early [21-23] and middle [24] childhood. Amniotic fluid samples from the Cambridge cohort were collected via amniocentesis during the second trimester of pregnancy (18 to 20 weeks gestation). One possible explanation for the null findings observed in the current study relates to the timing of testosterone exposure. Hormone concentrations are known to fluctuate throughout pregnancy [39], with considerable variability between measurements taken in the second and third trimesters [40]. In the current study, DNA sequencing confirmed that the cord blood samples were free from maternal blood contamination, and the higher concentrations of BioT in male compared to female offspring suggest that these samples reflect fetal circulation during late gestation. The lack of association between cord blood concentrations of BioT and autistic-like traits suggests that either prenatal testosterone does not influence these behaviors, or that any effects are at an earlier gestation.

It is also possible that the effects of prenatal testosterone exposure are determined not only by individual differences in concentration, but also by individual differences in biological sensitivity to testosterone. The neuroactive effects of testosterone occur either through the activation of the X chromosome linked androgen receptor AR gene, located at Xq11-12, or after aromatization to estradiol, through the estrogen receptor [41]. The cysteine, adenine, guanine (CAG) repeat sequence within exon 1 of the androgen receptor gene, is of particular interest because it is highly polymorphic. This CAG repeat codes for a polyglutamine tract of variable length in the N-terminal domain of the protein, and the number of repeats is inversely related to the transcriptional activity of androgen target genes [42,43]. It remains possible that testosterone levels during late gestation are associated with the ASD phenotype, but these effects are modified by individual genotype. However, it is important to note that studies of testosterone concentrations in both amniotic fluid [21-24] and cord-blood [29] have reported associations with postnatal behavior when naïve to individual genotype. We suggest that future studies in this area may benefit from an understanding of the genetic as well as endocrine background of an individual.

A further explanation for the null findings relates to the pattern of AQ scores observed in the current sample. The primary hypothesis of the current study – that perinatal testosterone concentrations are associated with autistic-like traits - was formulated based on widely observed sex-differences in ASD [6]. However, for the majority of AQ scales scored according to the original procedure [33], there were no differences in scores between males and females. This finding contrasts with studies from the Cambridge Fetal Testosterone Project, which have observed sex-differences in scores on other measures of autistic-like traits, including the Quantitate CHecklist for Autism in Toddlers among 18 to 24 month-old toddlers [23], and the Child Autism Spectrum Test and Autism-Spectrum Quotient-Child Version [24] among children between 6 and 10 years of age. It is possible that sample attrition may have contributed to the lack of sex differences in AQ scores, given that AQ scores were typically higher for socially disadvantaged participants, and that these individuals were less likely to take part in the current study. Importantly, however, sex differences were observed when an alternative scoring procedure of the AQ was applied [35], and these scales showed no association with early testosterone exposure in either sex.

Strengths of the current study include the relatively large sample size, the prospective longitudinal design spanning over two decades, and the highly sensitive and selective LC-MS/MS assay used to measure testosterone levels in umbilical cord blood. One potential concern is that degradation may have occurred in storage or with thawing, resulting in reduced concentrations of intact steroids. However, we believe such concerns are unfounded for several reasons. First, our sample set had been continually maintained at −80°C since collection and samples were thawed only once for aliquotting prior to shipping for assay. Studies of steroid stability (including testosterone) during long term storage confirm that serum samples are able to be stored for at least four decades at −80°C without loss or appreciable deterioration of steroid hormones [44,45]. Second, freshly collected cord blood samples run as quality controls (n = 5 to 6) had values within one standard deviation of the means of the frozen samples, adding further weight to the view that no significant degradation had taken place; our stability studies showed no effects of several freeze-thaw cycles. Finally, our recovery estimates based on spiking of fresh cord serum were 93% to 111% indicating that the assays did not suffer from masking or interference effects.

A limitation of the study design was that the ascertainment of the five ASD cases in this study was dependent upon parent-report of a clinician-based diagnosis (by a pediatrician, psychologist and speech pathologist) according to DSM-IV guidelines at any of the 5-, 8-, 10-, 14- or 17-year follow-ups. While it is commonplace in ASD research to confirm clinical diagnoses using ASD-specific behavioral observation and/or parent interview assessments, this was not possible within the Raine cohort. However, it is important to note that a previous investigation of direct observation data obtained prior to five years of age [46], found that each ASD case in the current study demonstrated behaviors consistent with ASD (for example, poor eye contact, delayed language, absence of pretend play). Moreover, the overall rate of ASD within the current sample was 0.71%, which is highly similar to the most recent population-based prevalence estimates in Australia (0.625%) [47], and suggests that there was no systematic bias introduced to the study by our reliance on clinician diagnosis.

A further limitation is that sample attrition in the current investigation, as for many longitudinal studies, appeared to bias the loss of individuals from lower socioeconomic strata. *Post hoc* analyses found that AQ scores varied according to maternal income and education, which raises the concern that the attrition may have underestimated any effect of maternal umbilical cord blood testosterone concentrations on AQ scores. However, published studies reporting significant associations between amniotic fluid testosterone levels and postnatal behavior [23,24], have included selected samples (that is, high-risk pregnancies undergoing amniocentesis) and experienced similar attrition effects. Furthermore, computer simulations using data from the Avon Longitudinal Study of Parents and Children (United Kingdom) have found that selective dropout in cohort studies only marginally affect regression coefficients, if participant selection occurs according to predictor variable(s) [48]. For these reasons, we suggest that the sample attrition in the current study had minimal, if any, influence on the null finings observed.

## Conclusions

The current study found no evidence that testosterone concentrations from umbilical cord blood are related to autistic-like traits in the general population. The findings suggest that any link between prenatal testosterone concentration and ASD may be restricted to exposure during the earlier stages of gestation, or due to individual differences in the biological sensitivity to testosterone.

## Abbreviations

AQ: Autism-spectrum quotient; ASD: Autism spectrum disorder; BioT: Bioavailable testosterone; CAG: Cysteine, adenine, guanine; CAH: Congenital adrenal hyperplasia; DSM-IV: Diagnostic and statistical manual of mental disorders, 4th edition; ELISA: Enzyme-linked immunosorbent assay; LC-MS/MS: Liquid chromatography-tandem mass spectrometry; SHBG: Steroid hormone binding globulin; TT: Total testosterone.

## Competing interests

The authors declare that they have no competing interests.

## Authors’ contributions

JAK undertook the analysis of the testosterone data. AJOW developed the hypotheses, conducted the statistical analyses, wrote the main drafts of the manuscript, and is responsible for correspondence and requests for reprints. All authors contributed to the interpretation and discussion of the results and have read and approved the final version of the manuscript.

## Supplementary Material

Additional file 1 Thresholds for ‘high’ scores on the various AQ scales in the current study. Click here for file
