# Supplementary material for: Perinatal testosterone exposure and autistic-like traits in the general population: a longitudinal pregnancy-cohort study
Source: J Neurodev Disord. 2012 Oct 30;4(1):25. doi: 10.1186/1866-1955-4-25 (PMC3500651; doi:10.1186/1866-1955-4-25)
Supplement: Additional file 1 — Thresholds for ‘high’ scores on the various AQ scales in the current study. [file 1866-1955-4-25-S1.doc]

Appendix A

Thresholds for ‘high’ scores on the various AQ scales in the current study

| Scale | Threshold for high score (upper decile) |
| --- | --- |
| AQ original scoring |  |
| Total | ≥ 22 |
| Social Skills | ≥ 5 |
| Communication | ≥ 5 |
| Attention Switching | ≥ 7 |
| Attention to Detail | ≥ 8 |
| Imagination | ≥ 5 |
| AQ alternate scoring |  |
| Total | ≥ 118 |
| Social Skills | ≥ 32 |
| Details/Patterns | ≥ 26 |
| Communication/Mindreading | ≥ 18 |
